# Identification, determination, and study of antioxidative activities of hesperetin and gallic acid in hydro-alcoholic extract from flowers of *Eriobotrya japonica* (Lindl.)

**Published:** 2014

**Authors:** Amir Hossein Esmaeili, Akbar Hajizadeh Moghaddam, Mohammad Javad Chaichi

**Affiliations:** 1 *Department of Laboratory Science, Babol-Branch, Islamic Azad University, Babol, I. R. Iran*; 2 *Faculty of Basic Science, University of Mazandaran, Babolsar, I. R. Iran*; 3 *Faculty of Chemistry, University of Mazandaran, Babolsar, I. R. Iran*

**Keywords:** *Antioxidant*, *Eriobotrya japonica Lindl*., *Flavonoids*, *Free radicals*, *Phenolic compounds*

## Abstract

**Objectives:** *Eriobotrya japonica* belongs to the Rosaceae. Studies have shown that the flowers of this plant are rich in phenolic and flavonoid compounds. Accorrdingly, the evaluation of antioxidative effects of *Eriobotrya japonica *Flower Extract (EJFE) have been performed *in vitro*.

**Material and Methods:** In this study, to investigate the influences of components of EJFE on its antioxidative activity, extract was prepared using hydro-alcoholic (25:75 V/V) solvent and the antioxidative activity of the extract was evaluated based on the scavenging of various radicals (DPPH and H_2_O_2_) by spectrophotometric method and chelating of ferrous ions by ferrozine reagent.

**Results:** HPLC analysis of the *Eriobotrya* *japonica *Flower Extract (EJFE) revealed hesperetin and gallic acid as the major antioxidants. When the content of total flavonoid and polyphenolic compounds in the flower extract of this plant was examined, a significantly higher level of total polyphenols was found in *Eriobotrya japonica *flower extract.

**Conclusion:** Results demonstrate that the high ability to scavenge free radicals, reducing power, and Fe^+2^chelating activity exerted by the EJFE were due to the high content of hesperetin and gallic acid in the flowers.

## Introduction

*Eriobotrya japonica* *(E. japonica *Lindl.*)* is a tree with edible fruit in the Rosaceae family, indigenous to southeastern China and Japan. Now it is also cultivated in the Mediterranean areas, Australia, South Africa, South America, California, India, and North of Iran. Its white flowers turn into pale-yellow or deep-orange pomes (Vaughan and Geissler, 1997 ▶). Its leaves have been used for treatment of skin diseases and diabetes (De Tommasi. et al, 1992 ▶), chronic bronchitis, coughs, phlegm, ulcers, and cancer (Ito et al., 2000 ▶). Reactive oxygen species (ROS) in the forms superoxide (^•^O_2_^-^), hydrogen peroxide (H_2_O_2_), and hydroxyl radical (^•^OH) are by-product of normal cellular metabolism and attack biological systems leading to destruction and peroxidation of cell membranes, many age-related disease, and susceptibility to cancer and infection (Yen and Chen, 1995 ▶). Flavonoids and phenolic compounds are secondary metabolites found to be abundant in the plant kingdom and effective in human health and decrease the risk of several diseases by reducing oxidative stress (Peluso, 2006 ▶). In this study, we report the separation of the main phenolic and flavonoid constituents from the flower extracts of *E. japonica*, which have not been previously reported. 

## Materials and Methods

Hesperetin was purchased from Sigma (Steinheim, Germany), Gallic acid from Acros (Geel, Belgium), and HPLC grade methanol from Fluka (Buchs, Switzerland). Ethanol and BHA were from Merck (Darmstadt, Germany). Napthylethylenediamide dihydrochloride, Sodium nitroprusside (SNP), Sulfanilamide, FeCl_2_, FeCl_3_, 1,1-Diphenyl-2-Picryl Hydrazyl radical (DPPH), 2,4,6-Tri- Pyridyl-S-Triazine (TPTZ), Butylated hydroxy anisole (BHA) and EDTA were purchased from Sigma-Aldrich (St. Louis, MO, USA). All Millipore syringe filters (0.22 and 0.45μm) were purchased from Millipore Company (JET BIOFIL^®^ Syringe Driven Filters). The double distilled water was used. Other chemicals were purchased from Sigma-Aldrich. The highest commercially available purity reagents were used.

**Preparation of the extract for HPLC**

Extraction procedure was performed according to the method proposed by Hertog et al. (1992) ▶. Briefly 0.5g of sample was refluxed using 50ml of 50% aqueous methanol at 90°C for two hours. This solution contains 1.5 g L^-1 ^of butylated hydroxy anisol(BHA**)** in order to prevent the analytes from oxidation. The obtained extracts were cooled, filtered to remove solid particles, then filtered by a 0.45 µm syringe Millipore filters and injected into the HPLC system.

**HPLC system**

The chromatographic measurements were carried out with HPLC system consisted of a model 515 solvent delivery system equipped with model 7725i injector fitted with a 20 μL loop. Column used was Spherisorb C18 (250×4.6 mm, 5 μm) all from Waters (Milford, MA, USA). The UV detector was model LC-95 set at 260 nm. The mobile phase used for separation and determination of analytes was methanol: 0.4% phosphoric acid (42.5 / 57.5 v/v) containing 0.5% THF as organic modifier with flow rate of 1.0 ml min^-1^ at 30 °C. Determination of gallic acid and hesperetin in EJFE was performed using standard addition method with HPLC at λ = 260 nm. Identification of each compound was performed by its retention time and spiking with the standard.

**Preparation of the extract for evaluation of antioxidant properties (in vitro)**

*E. Japonica* flowers were collected freshly from gardens of Ghaemshar, Mazandaran, Iran in october 2012. In outset, the flowers were exposed in the shadow for two weeks, then kept in 37ºC Oven for one day and were finally pulverized. About 81.5 grams of the flowers dry powder was shaken and extracted exhaustively with hydro-alcoholic (25:75 v/v). Next, it was filtered with Whatman filter paper and concentrated under reduced pressure in a rotary evaporator in 30 ºC for 6h to yield dried hydro-alcoholic extract, which was 9.5% of the dry weight of the plant flower. 

**Determination of total phenolic content in EJFE**

The amount of total phenolic compounds in extracts was determined spectrophotometrically using Folin-Ciocalteu Reagent(FCR) with small modifications (Singleton et al, 1999 ▶, Fukumoto and Mazza., 2000 ▶). One hundred milligrams of the extract was extracted with 10 ml of ethanol/water (75:25, v/v, 0.3% HCl) and filtered through a 0.22 μm Millipore filter. Two hundred and fifty microliter filtrate was mixed with 1.25ml of FCR (0.2 M) and 1ml of sodium carbonate (7.5 g dl^-1^). The mixture was incubated in the dark at room temperature for 2h to complete the reaction. Then, absorbance of the solution was measured at 760 nm with a T80^+^ UV-VIS spectrophotometer using distilled water as the blank. Evaluation was based on the standard curve of gallic acid (concentration range of 0-2 mg ml^-1^), which was dissolved in ethanol/water (75:25, v/v, 0.3% HCl). The concentration of total phenolic compounds was expressed in mg gallic acid equivalents (GAE) per gram of dried extract. All measurements were replicated three times.

**Estimation of total flavonoid content in EJFE**

Total flavonoid content in the extracts was determined spectrophotometrically using AlCl_3_ reagent (Jia et al., 1999 ▶) with minor changes. One hundred milligrams of the extract was extracted with 10 ml of ethanol/water (75:25 v/v, 0.3% HCl) and filtered through a 0.22 μm Millipore filter. 250 μL filtrate was mixed with 750 μL of methanol and 50 μL of AlCl_3_.6H_2_O (10% ethanolic) plus 50 μL of potassium acetate (1M) and 1.4 ml of double distilled water. After incubation at room temperature for 30 min, the absorbance of the reaction mixtures was measured at 415 nm. The blank sample was a 1:1 mixture of the examined extracts and double distilled water. Flavonoid content was expressed in mg hesperetin equivalents (HE) per gram of dried extract by using a standard curve of hesperetin(concentration range 0-1 mg ml^-1^). All measurements were replicated three times.

**Determination of reducing power in EJFE**

The reducing power of the *Eriobotrya japonica* Lindl. flower extract was determined according to Yen and Chen method (1995) ▶. In this method, about 0.5 ml of different amounts of the extract (25-800 μg ml^-1^) in water were mixed with phosphate buffer (1.25 ml, 0.2 M, pH 6.6) and potassium ferricyanide [K_3_Fe (CN)_6_] (1.25 ml, 1%). 

The mixtures were incubated for 20 minutes at 50°C. 1.25 ml of TCA (10%) was added to the mixture to stop the reaction, and then it was centrifuged at 2500g for 10 min. The supernatant (1.25 ml) was mixed with distilled water (1.25 ml) and FeCl_3_ (250 μL, 0.1%), and absorbance was measured at 700 nm against a blank solution. All measurements were replicated three times. Increased absorbance of the reaction mixture indicated increased reducing power. Ascorbic acid (Vit-C) was used as positive control.

**Metal chelating activity by EJFE**

Metal ions play an important role as catalysts of oxidative processes, leading to the formation of hydroxyl radicals by decomposing hydrogen peroxide. The chelating of ferrous ions by EJFE was estimated using ferrozine reagent (Dinis et al., 1994 ▶, Ebrahimzadeh et al., 2009 ▶) with minor modifications. One milliliter of the extract (concentration range of 10-100 μg ml^-1^) was added to a solution of 2 mM FeCl_2_ (0.05 ml). 

The reaction was initiated by addition of 5 mM ferrozine (1 ml), then it was shaken vigorously and left standing at room temperature for 10 min. Absorbance of the solution was then measured at 562 nm. The percentage inhibition of ferrozine- Fe^2+^ complex formation was calculated as [(A_b_-A_t_ or (As)/ A_b_] × 100, where A_b_ was the absorbance of the blank, A_t_ was the absorbance of the extract and A_s_ was the absorbance of positive control, EDTA.

**DPPH radical scavenging activity by EJFE**

The stable 1, 1-diphenyl-2-picryl hydrazyl radical (DPPH) scavenging ability in the flowers of the *E. japonica* was evaluated by spectrophotometric method (Yamaguchi et al., 1998 ▶). In this method, 1 ml of different concentrations of EJFE (concentration range of 0-60 mg ml^-1^) was added to a methanolic solution of DPPH (100 μM) at an equal volume. After 20 min at room temperature and in darkness, the absorbance was measured at 517 nm against a blank solution. The experiment was repeated three times. Vit-C, BHA and Hesperetin were used as standard controls. IC_50_ values denote the concentration of the sample which is required to scavenge 50% of DPPH free radicals.

**Scavenging of hydrogen peroxide by EJFE**

Scavenging of H_2_O_2_ was determined by the method of Ruch et al. (1989) ▶ with minor modification. A solution of hydrogen peroxide (40 mM) was prepared in phosphate buffer (pH 7.4). Approximately 2 ml the EJFE (concentration range of 0.1-1 mg ml^-1^) in methanol were added to a hydrogen peroxide solution (0.6 ml, 40 mM). The absorbance of hydrogen peroxide at 230 nm was determined after ten minutes against a blank solution containing phosphate buffer without H_2_O_2_. Ascorbic acid, BHA and Hesperetin were used as standards. The percentage of hydrogen peroxide scavenging by the EJFE and the standard compounds were calculated as follows: 

% scavenged (H_2_O_2_) = [(A_b_–A_t_ or A_s_)/ A_b_] × 100 where A_b_ absorbance of the control, A_t_ absorbance of the extract and A_s_ was the absorbance standard. The experiment was repeated three times.

## Results

**Figure 1:**
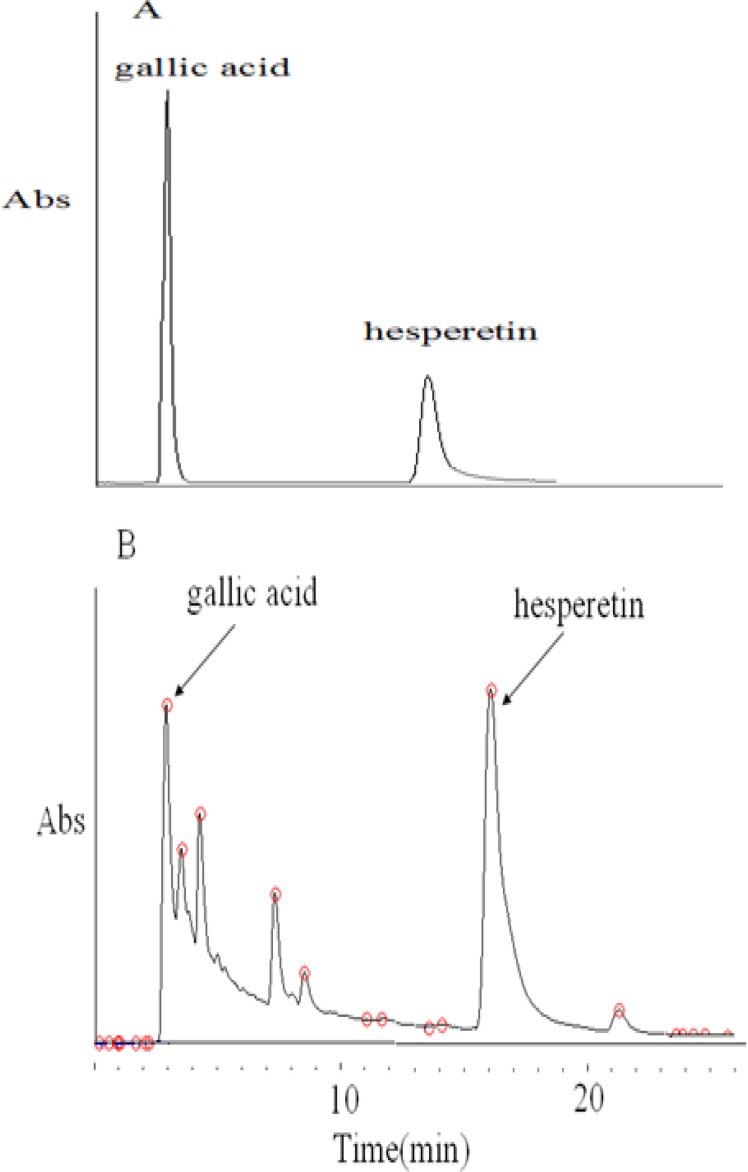
HPLC chromatogram of standards of gallic acid and hesperetin (A) and *Eriobotrya japonica* flower extract (EJFE) (B).

In our research, HPLC analysis of standards such as gallic acid, hesperetin (A) and *E. japonica* Extract (B) is shown in Figure 1. Several peaks were observed in the HPLC chromatogram. A major peak was obtained at a retention time of 3.4 min and was identified as gallic acid and other polar compounds. Another prominent peak was obtained at retention time of 16.6 and was identified as hesperetin (Figure 1). 

Statistical analysis was performed for validation of determination of analytes in *E. japonica.* Limit of detection (LOD) was calculated according to “3S_b_/m” where S_b _is the standard deviation of the blank and is equal to p-p Noise when only mobile phase was passing through the column for 45 minutes and “m” is the steepness of calibration curve. Linear dynamic range(LDR) was obtained by plotting the peak areas versus concentration and the relative standard deviation(RSD or %RSD) was determined by analyzing standard solutions (20 µg ml^-1^) repeated five times (Table 1).

**Table 1:** Statistical results for validation of determination of gallic acid and hesperetinin *Eriobotrya japonica*

**Compound**	**LOD(20μg ml** ^-1^ **)**	**LDR** **(20μg ml** ^-1^ **)**	**R** ^2^	**% RSD (n=5) **
**gallic acid**	0.07	0.07-35	0.999	1.22
**Hesperetin**	0.03	0.03-50	0.999	2.2

On the other hand, quantitative determination of total phenolic and flavonoids has shown that the EJFE contains 97.8 ± 0.7 mg g^-1^ of total polyphenols expressed as gallic acid equivalent (GAE, mg g^-1^ of extract) and 36.5± 0.3 mg g^-1^ of total flavonoids expressed as hesperetin equivalent. 

**Figure 2:**
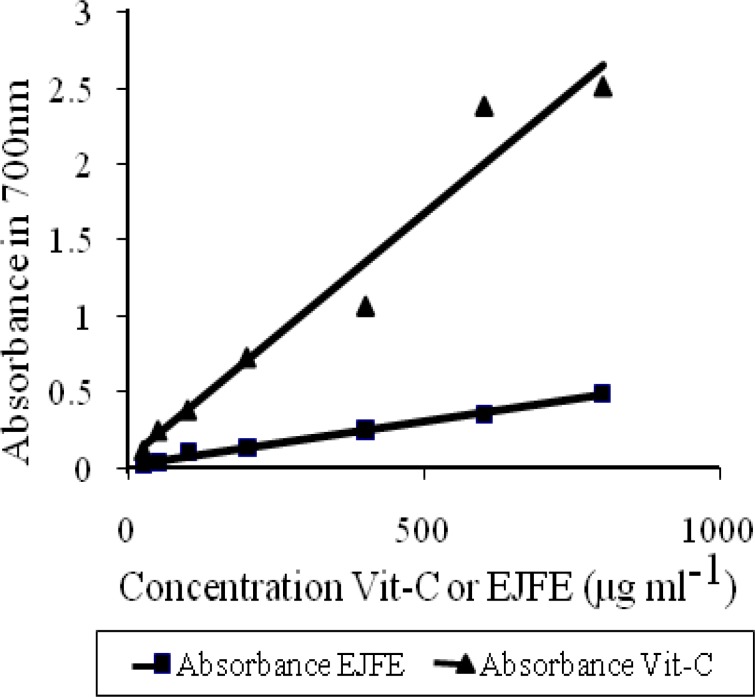
Reducing power of hydro-alcoholic extract of *Eriobotrya japonica* flower in comparison to Vitamine C (Vit-C). In concentration 800 μg ml^-1^, the reducing power of EJFE in comparison to Vit-C is lower about 19.52%. Each value is mean±SE. (n = 3).

Another part of the research evaluated the reducing power of EJFE in comparison to ascorbic acid (Vit-C). The reducing power of EJFE was found to be significant, but in comparison to Vit-C is lower dose-dependently (Figure 2).

**Figure 3:**
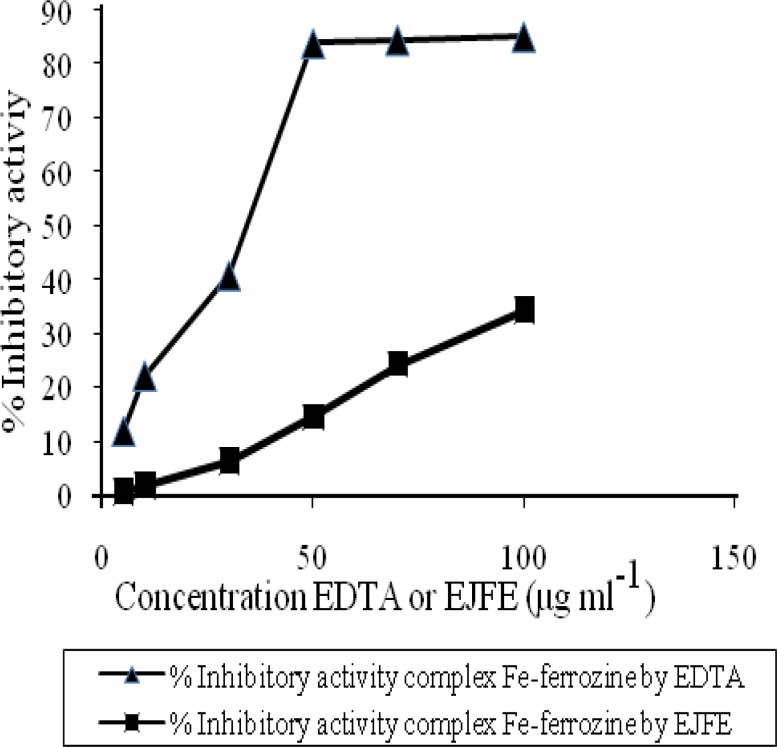
Chelating activity of EJFE in comparison to EDTA. The absorbance of the Fe^+2^-ferrozine complex decreased dose-dependently. In concentration 50 μg ml^-1^, chelating activity of EJFE in comparison to EDTA is lower about 17.29%. Each value is mean±SE. (n=3).

In experiments related to the chelating activities of the EJFE, Ferrozine can quantitatively form complexes with Fe^+2 ^to make a purple color. But, in the presence of other chelating agents such as plant extracts, the complex formation is reduced resulting in a decrease of the purple color of the complexes (Figure 3). 

The antioxidant activity of EJFE was evaluated in a series of in vitro tests. Each of these assays was based on one feature of antioxidant activity, such as the ability to scavenge various free radicals. In 20 mg ml^-1^ concentration, the EJFE significantly scavenged DPPH radicals in comparison to Vit-C, but less than BHA and Hesperetin (Table 2). Also, the neutralization of H_2_O_2_ by the *E. japonica *flower extract was measured spectrophotometrically. The ability of the assessed extract to neutralize H_2_O_2_ was dose dependent and it significantly scavenged H_2_O_2_ with the IC_50_ values of 3128.91 in 1000 μg ml^-1^ concentration (Table 3). 

**Table 2:** Percentage of neutralization of the DPPH radical by EJFE in comparison to Vit-C, hesperetin and BHA

**Concentration(20mg ml** ^-1^ **)**	**%Inhibitory activity DPPH(Mean±SD)**	**IC**_50 _ **(mg ml ** ^-l^ **)**
**Vit-C**	51.03±2.98	19.59
**Hesperetin**	63.81±2.76	15.76
**BHA**	86.77±4.31	11.52
**EJFE**	53.82±1.49	18.58

**Table 3:** Percentage of neutralization of H_2_O_2_ by EJFE in comparison to Vit-C,hesperetin and BHA

**Concentration (1000μg ml** ^-1^ **)**	**%Inhibitory activity H**_2_ **O**_2_ **(Mean±SD)**	**IC**_50 _ **(μgr ml** ^-1^ **)**
**Vit-C**	41.42± 6.54	1207.14
**Hesperetin**	19.51± 2.31	2562.78
**BHA**	24.57± 5.83	2035.01
**EJFE**	15.98± 3.66	3128.91

## Discussion

In this Study, the *E. japonica* extraction yield was about 9.5% of the dry weight of the plant flower with hydro-alcoholic extract (alcohol 96°). We performed HPLC analysis of EJFE to identify some of flavonoids and phenolic compounds and we showed that Hesperetin as bioflavonoid and gallic acid to be the major antioxidants in it. In quantitive determination of total polyphenols and flavonoids in EJFE, our study has shown that the extracts were the high contents of phenolic and flavonoid compounds. Based on the reports of Federico Ferreres et al. (2009) ▶, total phenolic content of *E. japonica* leaves, peel and flesh was about 64, 1337 and 1668 mg kg^-1^, respectively. Also, Lu et al. (2009) ▶ reported that total flavonoid content of leaves of *E .japonica* was 615 g kg^-1^, but our study showed that concentrations of gallic acid and hesperetin in the flower of this plant was 7200 and 15600 g kg^-1^, respectively. There is a relationship between antioxidant activities with the content of total phenolics or flavonoids. A high yield of polyphenols in the extract indicated that it possesses a high antioxidant activity.

In chelating activities tests, both plants extract and EDTA interfered with the formation of Fe^+2^ and ferrozine complexes, suggesting that it has chelating activity and captures ferrous ions before ferrozine. Chelation therapy may reduce iron-related complications in some diseases such as thalassemia major, cancer, HIV or Wilson’s disease (Grazul and Budzisz., 2009 ▶). 

Also, scavenging of H_2_O_2_ and DPPH by EJFE may be attributed to their phenolic compounds which can donate electrons to be, thus neutralizing it to water and neutral compounds. Since, H_2_O_2_ and free radicals can pass membranes and oxidize a number of cell compounds, thus elimination of hydrogen peroxide and DPPH, as well as the OH radicals is important for human health, food and drug. 

In this study, it was also described for the first time that EJFE has antioxidative activity through free radicals scavenging, metal chelatory, reducing power and detoxification.
